# Characterization of Common Chromosomal Translocations and Their Frequencies in Acute Myeloid Leukemia Patients of Northwest Iran

**DOI:** 10.22074/cellj.2016.3985

**Published:** 2016-04-04

**Authors:** Elnaz Amanollahi Kamaneh, Karim Shams Asenjan, Aliakbar Movassaghpour Akbari, Parvin Akbarzadeh Laleh, Hadi Chavoshi, Jamal Eivazi Ziaei, Alireza Nikanfar, Iraj Asvadi Kermani, Ali Esfahani

**Affiliations:** 1 Hematology and Oncology Research Center, Tabriz University of Medical Sciences, Tabriz, Iran; 2 Department of Pharmaceutical Biotechnology, Faculty of Pharmacy, Tabriz University of Medical Sciences, Tabriz, Iran

**Keywords:** Chromosomal Translocation, Acute Myeloid Leukemia, Iran

## Abstract

**Objective:**

Detection of chromosomal translocations has an important role in diagnosis and treatment of hematological disorders. We aimed to evaluate the 46 new cases of de novo acute myeloid leukemia (AML) patients for common translocations and to assess the effect of geographic and ethnic differences on their frequencies.

**Materials and Methods:**

In this descriptive study, reverse transcriptase-polymerase chain reaction (RT-PCR) was used on 46 fresh bone marrow or peripheral blood samples to detect translocations t (8; 21), t (15; 17), t (9; 11) and inv (16). Patients were classified using the French-American-British (FAB) criteria in to eight sub-groups (M0-M7). Immunophenotyping and biochemical test results of patients were compared with RT-PCR results.

**Results:**

Our patients were relatively young with a mean age of 44 years. AML was relatively predominant in female patients (54.3%) and most of patients belonged to AML-M2. Translocation t (8; 21) had the highest frequency (13%) and t (15; 17) with 2.7% incidence was the second most frequent. CD19 as an immunophenotypic marker was at a relatively high frequency (50%) in cases with t (8; 21), and patients with this translocation had a specific immunophenotypic pattern of complete expression of CD45, CD38, CD34, CD33 and HLA-DR.

**Conclusion:**

Similarities and differences of results in Iran with different parts of the world can be explained with ethnic and geographic factors in characterizations of AML. Recognition of these factors especially in other comprehensive studies may aid better diagnosis and management of this disease.

## Introduction

Chromosomal translocations have a significant role in the initiation of carcinogenesis by creating gene fusions that are causal for approximately 20% of human cancers (1, 2). So far, many gene fusions have been recognized that have important diagnostic and prognostic roles in malignant hematological disorders of which some are leukemia-associated markers for minimal residual disease (MRD) detection (2-4). Acute myeloid leukemia (AML) constitutes less than 1% of all cancers and 25% of all leukemia cases. It is more common in adults and its prevalence increases with age (5).

It was estimated that among 52,380 new cases of leukemia in the United States in 2014, 18,860 (36%) of them were AML cases, and among 24,090 estimated leukemia deaths, 10, 460 (43%) instances were due to AML (6). Balanced chromosomal rearrangements, in particular translocations, occur in 25 to 30% of AML cases. Because of their importance in recognizing genes involved in leukemogenesis and their relation with the treatment of patients, it has received much attention (7).

In AML, gene fusions often encode specific oncofusion proteins. Four most common rearrangements in AML include t (15; 17), t (8; 21), inv (16) and 11q23/MLL and have frequencies between 3 and 10%. These translocations respectively encode *PML-RARA, AML1-ETO, CBFB-MYH11* and *MLL-fusions* oncofusion proteins (8). Translocation t (8; 21) has a close relation with the AML-M2 subgroup in FAB classification and is mostly present in patients in this subgroup and rarely in M1 and M4 subgroups (9). The t (15; 17) translocation is found in about 95% of acute promyelocytic leukemia (APL), which is treatable in its early phase with all-trans retinoic acid (ATRA) (8, 10, 11).

Molecular cytogenetic analysis, compared with classical cytogenetic analysis, has many advantages including rapid and comprehensive detection of known target translocations. Reverse transcriptase- polymerase chain reaction (RT-PCR) is a fast and sensitive technique that can be used on small samples with low quality (12-15).

Given the importance of knowing the prevalence of chromosomal aberrations and their specific phenotypes in certain geographic region or ethnicity, for rapid diagnosis and best treatment selection, we aimed to evaluate the frequency of four common chromosomal translocations among 46 de novo AML patients.

## Materials and Methods

### Patient selection

In this descriptive study, 46 new cases of adult de novo AML who were diagnosed in Shahid Ghazi hospital (Tabriz, Iran) from 2012-2014 were included. AML diagnosis was confirmed by bone marrow aspiration and peripheral blood smears, total blood count, cytochemistry and immunophenotyping. Two independent oncologists classified patients based on the French-American-British (FAB) Cooperative Group criteria in eight subtypes (M0-M7). Cases with past clinical history and those who had received any treatment were excluded.

### Bone marrow aspiration collection

Aspiration specimens were collected in tubes with Ethylenediaminetetraacetic acid (EDTA, Merck, Germany) anticoagulant and transferred to the laboratory at 4˚C within 8 hours. In three cases in which aspiration was impossible, on the condition that we had enough blast cells, peripheral blood samples were collected.

### Mononuclear cell isolation

Mononuclear cells were isolated within 24 hours after sample collection. The white blood cell (WBC) count was adjusted to less than 20×10^3^ /ml by diluting specimens in phosphate-buffered saline (PBS, Sigma, USA). For cell isolation, we used Ficol (Baharafshan, Iran) and after collection of the mononuclear cell layer, cells were washed with 10 ml PBS containing 10% fetal bovine serum (FBS, Gibco, USA). The supernatant was removed after centrifugation (Sigma, USA), and 1 ml of Qiazol (Qiagen, USA) was then added to the cell precipitate to dissolve cells completely. This solution was stored in -70˚C, until RNA extraction.

### Total RNA extraction

Frozen samples were thawed at room temperature and mixed. Next 200 μl cold chloroform (Merck, Germany) was added to 1 ml of this solution and mixed and incubated in room temperature for 2 minutes. The solution was then centrifuged (Sigma, USA) at 4˚C and 18000 rpm for 30 minutes.

Fourto five hundred micro liter of aqueous phase was transferred into another microtube on ice and then 500-600 μl cold isopropanol (100%) (Merck, Germany) was added. After mixing, the solution was incubated on ice for 10-15 minutes and then centrifuged at 4˚C and 18000 rpm for 20 minutes.

After removing the supernatant, 0.5-1 ml, cold ethanol (Scharlau, Sentmenat, Spain) (75% in DEPC-treated water) was added to the precipitate and agitated gently. Finally it was centrifuged at 4˚C and 18000 rpm for 5 minutes. This washing process was repeated to acquire best results.

Then supernatant was discarded gently and microtubes were placed at room temperature to dry the RNA. Afterwards, microtubes were placed on ice and 50 μl DEPC-treated water (CinnaGen, Iran) was added to them with mixing. Pico drop (Pico drop Ltd, UK) was used to estimate the RNA concentration. Integrity of isolated RNA was analyzed indirectly by the quality of synthesized complementary DNA (cDNA).

### cDNA synthesis

Reverse transcription reaction was done according to the BioRT cDNA first strand synthesis kit protocol (Bioer Technology, Japan).

### Reverse transcriptase-polymerase chain reaction analysis

Primers used for the four common fusion transcripts of chromosomal translocations are given in Table 1. To assess presence of *AML1-ETO, PML-RARA* and *CBFB-MYH11*, final volume of PCR was 10 μl with 4 μl Master Mix, 4.5 μl dH_2_O, 0.5 μl cDNA and 0.5 μl of each primer (20 pmol/μl). PCR conditions were an initial denaturation step at 94˚C for 3 minutes, followed by 35 cycles at 94˚C for 45 seconds, 63˚C for 1 minute and 72˚C for 1.5 minutes. Final extension step was 72˚C for 7 minutes (16).

For the MLL-AF9 fusion transcript, all quantities remained the same. In this group initial denaturation step was at 94˚C for 5 minutes, followed by 35 cycles at 94˚C for 1 minute, 60˚C for 1 minute and 72˚C for 1 minute. The time of the final extension step also increased to 10 minutes (17).

The PCR products were analyzed on 2% agarose gel electrophoresis. We used confirmed positive patients samples from another source as positive controls. The blank control without cDNA was used in each run.

### Immunophenotyping

Specimens (bone marrow aspiration or peripheral blood) were collected separately in tubes with EDTA for immunophenotyping. After cell counting with the automatic analyzer (H1, Tecknicon, USA), cells were washed with PBS twice and cell count was then adjusted to 10-20×10^3^ /ml.

Our samples were first analyzed by a flow cytometer (BD FACS Calibur, Becton Dickinson, USA). Samples were then stained directly with fluorochrome-conjugated antibodies (DAKO, Denmark). We used the following antibodies according to the applied protocol (BD FACS): CD45-FITC for gating strategies, CD13-PE, CD14-PE, CD15-FITC, CD33-PE, CD41-FITC, CD 117-PE and GpA-FITC for myeloid line-specific antigens, CD2-PE, CD3-FITC, CD7-FITC, CD19-PE, CD20-FITC and CD22-PE for lymphoid line-specific antigens, CD34-PE, CD38-PE, HLA-DR-FITC and CD10-FITC for determination of maturation stage and CD11b-FITC for non-line-specific antigen. Data were analyzed with Cell quest (BD, USA), and positivity threshold for each monoclonal antibody was defined as 20% labeled cells.

### Biochemical analysis

We obtained biochemical test results of each patient that is undertaken routinely for all leukemia patients with an auto analyzer (Alesion, Abbott, Germany).

**Table 1:** Primers for *AML1-ETO, PML-RARA, CBFB-MYH11* and *MLL-AF9* fusion genes


Fusion transcripts	Primers 5´→3´	Size of PCR products(bp)

*AML1*	CTACCGCAGCCATGAAGAACC	395
*ETO*	AGAGGAAGGCCCATTGCTGAA	
*PML*	CAGTGTACGCCTTCTCCATCA	381
*RARA*	GCTTGTAGATGCGGGGTAGA	
*CBFB*	GCAGGCAAGGTATATTTGAAGG	418
*MYH11*	TCCTCTTCTCCTCATTCTGCTC	
*MLL(3920U)*	CTCAGCCACCTACTACAGGAC	852
*AF9(1645L)*	AGCGAGCAAAGATCAAAATC	


PCR; Polymerase chain reaction.

### Statistical analysis

SPSS version 21 (IBM, USA) was used for all statistical analysis. Descriptive statistics were used to describe the variables. Independent t test was used for analysis of relationship between biochemical results and prevalence of t (8; 21). A P value less than 0.05 was considered statistically significant.

### Ethical consideration

The Ethics Committee of the Tabriz University of Medical Sciences approved this study. Samples were collected after obtaining informed consent from each patient.

## Results

### Frequency of the four common chromosomal translocations

**Fig.1:**
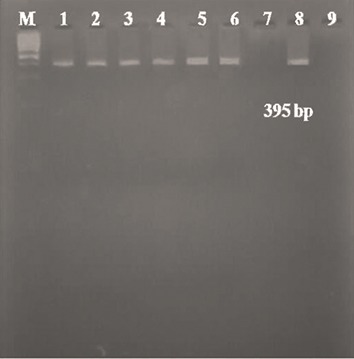
Ethidium bromide-stained agarose gel (2%) of PCR product for the *AML1-ETO* fusion gene (395 bp) with RT-PCR. Lane M; 100 bp ladder, Lane 1-6; Positive cases, Lane 7; Negative control, Lane 8; Positive patient control, Lane 9; Water blank and RT-PCR; Reverse transcriptase-polymerase chain reaction.

**Fig.2:**
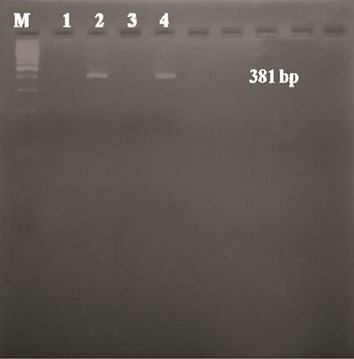
Ethidium bromide-stained agarose gel (2%) of PCR product for the *PML-RARA* fusion gene (381 bp) with RT-PCR. Lane M; 100 bp ladder, Lane 1; Negative control, Lane 2; Positive case, lane 3; Water control, Lane 4; Positive patient control and RT-PCR; Reverse transcriptase-polymerase chain reaction.

Among the 46 patients, six patients (13%) were positive for *AML1-ETO* (Fig.1) and one (2.7%) for *PML-RARA* (Fig.2). Other fusion transcripts (*CBFB-MYH11* and *MLL-AF9*) were absent in all patients (data was not shown).

### Clinical characteristics

Summary of clinical, hematological and phenotypic characteristics of all AML patients are reported in Table 2 and specific characteristics of t (8; 21) positive patients are reported in Table 3.

Recently morphologic FAB classification of four t (8; 21) positive patients revised and approved again, but for the other two patients, smears were not available.

The patient who was positive for t (15; 17) was a non-smoking 44 year-old male. His FAB classification was M3 with B positive blood group. His white blood cell count was 33.84×10^3^ /μl and his platelet count was 21×10^3^ /μl with 4.9 g/dl hemoglobin and 20% blasts in the first peripheral blood test.

### Immunophenotypic characteristics

Frequency of some immunophenotypic antigens in the total AML sample set were: CD45: 100%, CD33: 97.9%, CD38: 95.7%, CD13: 82.6%, HLA-DR: 65.2%, CD117: 63%, CD11b: 60.9%, CD34: 54.3%, CD14: 15.2%, CD15: 28.3% GpA: 6.5% and CD19: 8.7%.

Immunophenotypic information for patients with t (8; 21) are given in Table 4. Our t (15, 17) positive patient had the following immunophenotypic characteristics: CD13: 80%, CD14: 39%, CD33: 76%, CD11b: 27%, CD45: 94% and CD38: 76% with all other (especially CD34 and HLA-DR) being negative.

### Expression of CD19 at t (8; 21)

The relation between CD19 expression and t (8; 21) has previously been reported (18, 19). Expression of CD19 in our t (8; 21) positive patients was 50% but it showed 2.5% expression in patients that were negative for this translocation. However, since the number of positive patients was small, this finding was not statistically significant.

### Biochemical results of positive translocation patients

Results of common biochemical tests for t (8; 21) positive patients are reported in Table 5. We observed no relation between these results and the occurrence of translocation.

**Table 2:** Summary of clinical, hematologic and morphologic characteristics for total acute myeloid leukemia patients


	n(%)	Mean (SD)	Range (minimum-maximum)

Age		44 (16.59)	64 (16-80)
Male/Female	21 (45.7%)/25 (54.3%)		
FAB	M0 1 (2.2%)		
	M1 5 (10.9%)		
	M2 16 (34.7%)		
	M3 4 (8.7%)		
	M4 8 (17.4%)		
	M5 4 (8.7%)		
	M7 1 (2.2%)		
	Unknown 7 (15.2%)		
	Total 46 (100%)		
WBC (×10^3^ /μl)		35.65 (49.19)	201.43 (0.57-202)
Hb (g/dl)		8.5 (1.68)	6.7 (4.9-11.6)
PLT (×10^3^ /μl)		73.08 (92.97)	493 (12-505)
Blast (%)		28.14 (20.03)	85 (3-88)


FAB; French-American-British type, WBC; White blood cell, Hb; Hemoglobin and PLT; Platelet .

**Table 3:** Clinical, hematologic and morphologic characteristics for six patients with t (8; 21) positive acute myeloid leukemia


Case number	1	2	3	4	5	6

Sex	M	F	F	M	M	M
Age	25	33	16	58	59	54
Familial history	N	N	N	N	N	N
Smoking	N	N	N	N	Pos	N
WBC (×10^3^ /µl)	36.12	5.64	6.81	55.75	8.58	12.67
PLT (×10^3^ /µl)	14	17	14	12	26	14
Hb (g/dl)	6.3	9.4	10.1	9	7.4	8
Blast (%)	19	28	33	56	32	45
Blood group	A+	AB+	O+	O+	A+	A+
FAB	M2	M2	M2	M2	M4	M2


M; Male, F; Female, N; Negative, Pos; Positive, WBC; White blood cell, Hb; Hemoglobin, PLT; Platelet and FAB; French-American-British type.

**Table 4:** Immunophenotypic analysis of six patients with t (8; 21) positive acute myeloid leukemia*


Case number	1	2	3	4	5	6

CD45	91	92	95	98	98	99
CD38	90	90	98	98	86	100
CD33	81	74	65	82	89	50
CD34	70	61	88	47	31	71
HLA-DR	35	79	95	50	52	94
CD13	N	13	85	83	45	96
CD117	70	73	88	44	N	N
CD11b	38	15	18	32	69	6
CD15	N	8	N	20	96	N
CD19	48	N	N	42	N	97
CD14	9	N	N	7	11	N
GpA	N	N	N	N	N	N
CD41	N	N	N	N	N	N
CD10	N	N	N	N	N	N
CD7	N	N	N	N	N	N
CD3	N	N	N	N	N	N
CD2	N	N	N	N	N	N
CD22	N	N	N	N	N	N
CD20	N	N	N	N	N	N


GPA; Glycophorin A, N; Negative and *; Data are given as percentages.

**Table 5:** Biochemical test results for six patients with t (8; 21) positive acute myeloid leukemia


Case number	1	2	3	4	5	6

LDH (U/L)	4160	ND	728	2437	ND	1894
Urea (mg/dl)	32	28	21.4	39	28.1	34
Creatinin (mg/dl)	ND	0.39	0.43	0.96	0.85	1.29
SGOT (U/L)	144	27	14	43	13	22
SGPT (U/L)	254	91	10	37	183	15
ALP (U/L)	ND	151	224	165	ND	148
Uric Acid (mg/dl)	6.2	ND	2.4	4.5	ND	6.8
FBS (mg/dl)	117	ND	ND	ND	98	114


LDH; Lactate dehydrogenase, SGOT; Serum glutamic oxaloacetic transaminase, SGPT; Serum glutamic-pyruvic transaminase, ALP; Alkaline phosphatase, FBS; Fasting blood sugar and ND; Not done.

## Discussion

Acute myeloid leukemia is a clonal heterogeneous disorder of hematopoietic progenitor cells that is most common in adults (20). Presence of recurrent chromosome abnormalities alone, such as t (8; 21), t (15; 17) and inv (16), is sufficient to diagnose AML (21). Diagnosis of chromosomal abnormalities may help to recognize cause of leukemogenesis and provide new strategies for treatment of patients (22). Geographic differences of chromosomal abnormalities in hematological disorders have been previously described (23, 24). In one report annual incidence of leukemia in Tabriz (largest city in Northwest of Iran) was 3.7 per 100,000 and incidence of AML in Northwest of Iran was 1.37 per 100,000 (25). There is a lack of information about the cytogenetic patterns of AML patients from many parts of the world and even in Iran where cytogenetic distribution in various ethnicities in different regions is unknown.

In this study, mean age of de novo AML patients was 44 years which is relatively similar to that of Malaysian patients (39 years) (26) but different to that in western countries (71 years) (27). The frequency of AML M2 (34.7%) was higher than any other subgroup and its frequency is comparable to that in Germany (39.6%) (28), USA (37%) (29), China (29.9%) (30), Taiwan (53%) (31), Korea (48.3%) (32), Hong Kong (50%) (33) and Malaysia (33.3%) (34). After that, M4 and M1 were the most frequent with 17.4 and 10.9% of patients respectively. AML M4 had a lower frequency than Germany (20.4%) (28) and USA (23%) (29) but a higher frequency compared with China (5.3%) (30), Taiwan (10%) (31), and Hong Kong (13.3%) (33). AML M3 (8.7%) has a relatively higher frequency compared with Germany (5%) (28) and USA (2%) (29), and it is lower than China (25.3%) (30), Taiwan (20%) (31), Korea (20.7%) (32), Hong Kong (20%) (33).

In a study of patients in Northeast of Iran, M4 had the highest frequency (24.58%), and then M1 (20.67%) M2 (17.88%) and M3(16.76%) had the highest frequencies (35). However, in another study in Iran (based in Tehran), results were different with M2=34%, M3=33%, M1=24% and M4=5% (36).

AML distribution in our female patients (54.3%) was relatively higher than males. Translocation t (8; 21) has 13% frequency among AML patients. This is higher than Malaysia (7.5%) (26), China (8.3%) (30), USA (6%) (29), German study (4.3%) (28) and Northeast of Iran (8.9%) (35) but lower than Tehran-Iran (25.9%) (36), Korea (34.5%) (32) and Taiwan (23%) (31). Frequency of t (8; 21) in our M2 subgroup was 31.2% and comparable with Japan (33.1%) (23) and Malaysia (37.5%) (34) but higher than Australia (15.3%) (23) and Hong Kong (13.3%) (33). This frequency was lower than that of Northeast of Iran (50%) (35), Tehran-Iran (75%) (36) and Taiwan (43.7%) (31). All these different presentations of t (8; 21) may be due to different ethnicities in different geographic regions but more research with high number of patients is needed. The age of translocation t (8; 21) positive patients was under 60 years which is similar to most studies like in China and America. Moreover, all of these patients had anemia and thrombocytopenia.

Translocation t (15; 17) had a lower frequency (2.17%) and this is comparable to Malaysia (2.3%) (26) and USA (7%) (29) but lower than China (14.3%) (30) and Tehran-Iran (27%) (36). We had some limitations in confirming our M3 FAB classification, because bone marrow aspiration was impossible in some of them, even for immunophenotyping. Some patients expired very soon even in the first week. In some patients, blood or bone marrow smears were not available for revision. Finally in some of them immunophenotype, morphology and pathology results were contradictory. Because of these limitations, further comprehensive studies with all variant translocations in this subgroup are essential.

Results for inv (16) and t (9; 11) were negative in our study and this is may be because of low frequency of these abnormalities around the world and for better investigation, higher number of cases is needed (7, 8).

All of our patients were positive for the CD45 immunophenotypic marker, and myeloid specific markers (CD13 and CD33) were present with high percentage. All our t (8; 21) positive patients had a specific immunophenotypic pattern and strongly expressed CD45, CD38, CD34, CD33 and HLA-DR with 100% frequency. Among aberrant antigens, CD19 was expressed with 50% frequency. This is comparable with that in Australia (57.1%) and Japan (71.9%) (23). This is also in accordance with studies that have established expression of B cell linage genes such as CD19 and PAX5 as hallmarks of t (8; 21) (37).

## Conclusion

We show that similarities and differences with other studies around the world such as age of our patients, high frequency of AML M2 and M4, relatively higher female rate of patients and common incidence of t (8; 21), apart from our small sample size, could be evidence of ethnic or geographic factors on different patterns of leukemia patients. Immunophenotypic results of our t (8; 21) positive patients had complete specific expression of CD45, CD38, CD34, CD33 and HLA-DR with 50% expression of CD19. This may help rapid decision making of cytogenetic analysis selection for these patients in the future. However, comprehensive cohort studies with higher numbers of patients with more detailed translocation analysis are recommended.
